# Knowledge gaps in food allergy among the general public in Jeddah, Saudi Arabia: Insights based on the Chicago food allergy research survey

**DOI:** 10.3389/falgy.2022.1002694

**Published:** 2022-12-23

**Authors:** Abdulrahman Ahmad Takrouni, Ibrahim Omer, Faisal Alasmari, Suhayb Islamuldeen, Amr Yasser Ghazzawi, Mohammed Ibrahim Zahrani, Mohamed Eldigire Ahmed, Amir Abushouk

**Affiliations:** ^1^ College of Medicine, King Saud bin Abdulaziz University for Health Sciences, Jeddah, Saudi Arabia; ^2^ King Abdullah International Medical Research Center, Jeddah, Saudi Arabia;; ^3^ Ministry of the National Guard-Health Affairs, Jeddah, Saudi Arabia; ^4^ College of Science and Health Professions, King Saud bin Abdulaziz University for Health Sciences, Jeddah, Saudi Arabia

**Keywords:** knowledge, attitudes, food allergy, igE-mediated food allergy, food-induced anaphylaxis, food intolerance, general public, Jeddah - Saudi Arabia

## Abstract

**Background:**

Food allergy is an increasing health concern. Studies have shown that food allergy knowledge is lacking among people, especially in areas related to distinction between food allergy and intolerance, symptoms recognition, and current means of management. This knowledge gap puts allergic patients at more risk of getting fatal anaphylactic reactions, which occur mostly in public areas. Locally, Public's knowledge and attitudes of food allergy was not sufficiently investigated. Therefore, we aim to assess food allergy knowledge and attitudes among Jeddah population in Saudi Arabia.

**Methods:**

We adopted The Chicago Food Allergy Research Survey for the General Public (CFARS-GP) and used it as a data collection tool. We hosted the questionnaire on Google Forms and distributed the link through social media outlets targeting individuals of Jeddah population who are 18 years old and above.

**Results:**

A total of 510 individuals completed the survey. The respondents answered 56% of the knowledge-based items correctly. Knowledge was strongest in symptoms/severity and definition/diagnosis, while it was weakest in susceptibility and prevalence, distinction between food allergy and intolerance, and food allergy management. Higher knowledge was significantly associated with prior training in food allergy, food-allergic acquaintance (i.e., having food allergy or knowing an allergic patient), and being a relative of a health care worker. For the attitudes, respondents thought that food allergy negatively affects patients' quality of life, and that schools should establish policies to protect allergic children; however, they downplayed stigma associated with food allergy.

**Conclusion:**

Increased food allergy knowledge among the general public is needed especially in areas related to susceptibility and prevalence, distinction between food allergy and intolerance, triggers and environmental risks, and the management of food allergy. Prior experience with food allergy through (1) training, (2) food-allergic acquaintance, or (3) being a relative of a health care worker increases food allergy knowledge significantly. Thus, targeted educational interventions might have a significant effect in improving food allergy knowledge among the general population.

## Introduction

Food Allergy is defined as an immune-mediated response that is initiated by IgE antibodies upon exposure to a given food allergen (1). Food allergy has been known to be more prevalent among the pediatric population. Osborne and colleagues found that 10% of 1 year old infants in Australia have IgE-mediated food allergy that is proven by oral food challenge (2). Furthermore, it was reported that one in every twelve children in the United States has IgE-mediated food allergy (3). However, an increase in the prevalence of this condition has been reported among adults as well. For instance, a cross-sectional study in the United States estimated that one out of every ten adults has IgE-mediated food allergy (4).

Food is the most common trigger of anaphylaxis, an acute life-threatening systemic allergic reaction (1, 5). Death is a rare outcome of food-induced anaphylaxis that is caused mainly by accidental exposures to allergens. Additionally, poor management of food-induced anaphylactic reactions is a contributing factor to fatality. Several studies indicated that the incidence rate of fatal food anaphylaxis among the general population was low (0.03–0.3 deaths per million person per year), and that delayed use of intramuscular epinephrine injections has resulted in fatality in most of these cases (6–8).

Moreover, Pouessel and colleagues found that fatal anaphylactic reactions occur primarily following an unintentional exposure to food allergens outside home, such as in restaurants and schools (9). Thus, in order to deal with such cases, members of the general population should have sufficient food allergy knowledge, especially in management and epinephrine injections use. Despite the important role of the general population in the management of food-induced anaphylaxis, global and local data suggest that their food allergy knowledge is lacking. Gupta and et al. assessed food allergy knowledge among the US population and found that knowledge was weakest in areas related to current means of treating food allergy (10). Similarly, data from the limited number of available local studies suggest that there is an inadequate food allergy knowledge among the general public in Saudi Arabia with management being one of the major areas of poor knowledge (11, 12).

Poor food allergy knowledge among members of the general population, especially in areas related to symptoms recognition and current means of management, increases the risk of mortality among allergic individuals because they commonly experience anaphylactic reactions in public areas. Also, people who cannot differentiate between food allergy and other food related adverse reactions might label themselves as allergic patients and unnecessarily avoid some food. Therefore, the primary aim of this study is to assess the general public's knowledge and attitudes toward food allergy in Jeddah, Saudi Arabia.

## Materials and methods

### Study design

This is a cross sectional study that collected data using convenience sampling *via* an online questionnaire in both Arabic and English versions. We hosted the questionnaire on Google Forms webpage and distributed the link through social media channels targeting individuals of the general population of Jeddah city (aged 18 years and above) from May to August 2021. Participation was voluntary, and we administered an informed consent alongside the questionnaire. It is estimated that 3,457,794 individuals currently reside in the city of Jeddah (13). We calculated the targeted sample size considering the following criteria: (1) 95% confidence level; (2) 5% margin of error; and (3) 50% response rate and had resulted in a sample of 385 individuals. The institutional review board of King Abdullah International Medical Research Center (KAIMRC) approved this study. (approval No: JED-21-427780-49687).

### Study instrument

A questionnaire of 47 items divided into 3 sections was used as a data collection tool (Supplementary Survey). The first section collected demographical characteristics of the participants (e.g., age and gender) using 11 items. In one of the questions in this section, an answer option was accidentally deleted from the Arabic version of the online survey that was distributed to the participants (Supplementary Table S1). The second section assessed the participants' knowledge of food allergy *via* a 17-item quiz (14 true/false and 3 multiple choice questions) which consists of 5 domains: definition and diagnosis, symptoms and severity, triggers and environmental risks, susceptibility and prevalence, and treatment and use of health care. For example, the participants were asked whether milk intolerance is the same as food allergy, and if lactating mothers could pass allergens to their babies through breast feeding. The third section assessed the attitudes and beliefs of the participants towards food allergy using 19 items (13 5-point Likert-scale with response options ranging from1 = Strongly Disagree to 5 = Strongly Agree, 4 multiple choice, and 2 Dichotomous styled questions with Yes/No response options). This section assessed the participants' attitudes towards stigma and acceptability, quality of life, current means of treatment and management, and policy issues related to this condition. For instance, we asked the participants if they agree that food allergy is a serious health concern locally, and that food allergy patients can eat safely at restaurants. Both the second and third sections were adopted from the Chicago Food Allergy Research Survey for General Public (CFARS-GP) developed by Gupta and colleagues (14). Minor modifications were made on the questionnaire to meet the study criteria. Forward and backward translations followed by an extensive review were made to ensure the quality of the translated version. With regard to the survey translation, a group member has translated the original survey into the Arabic language (forward translation). Then another group member who was blinded to the original survey has translated the Arabic version back into English (backward translation). The new English version was then compared with the original survey, and adjustments were made accordingly. This process was done to avoid literal translation and to ensure that all respondents perceive the questions in the same way regardless of the language they use to fill the survey.

### Data analysis

All the collected data were stored in an excel sheet. We cleaned the data by screening the Excel sheet manually and excluding both incomplete responses and responses that did not meet the inclusion criteria. In addition, all data entry mistakes were identified and corrected. With regards to outliers, some were identified in the knowledge scores of respondents, but they were not deleted because they did not influence the data significantly. In addition, we coded the data with the same order of options that appears in the questionnaire. Both the cleaning and coding were done prior to the analysis. We used JMP®, Version 15. SAS Institute Inc., Cary, NC, 1989–2021 in the analysis. Data were described by frequency and percentage distributions. We calculated mean and standard deviation for scale items. We used independent samples t-test to compare the mean knowledge score between 2 independent groups. Additionally, we used Analysis of Variance (ANOVA) to compare the mean knowledge score between three or more independent groups. We used independent samples t-test and ANOVA because the data were normally distributed. The overall knowledge score was defined as the percentage of each respondent's true answers out of total items in the knowledge component. Both false and I don't know responses were considered as not true answers. A *P* value less than 0.05 was considered significant.

## Results

### Participant's characteristics

**Figure 1:**
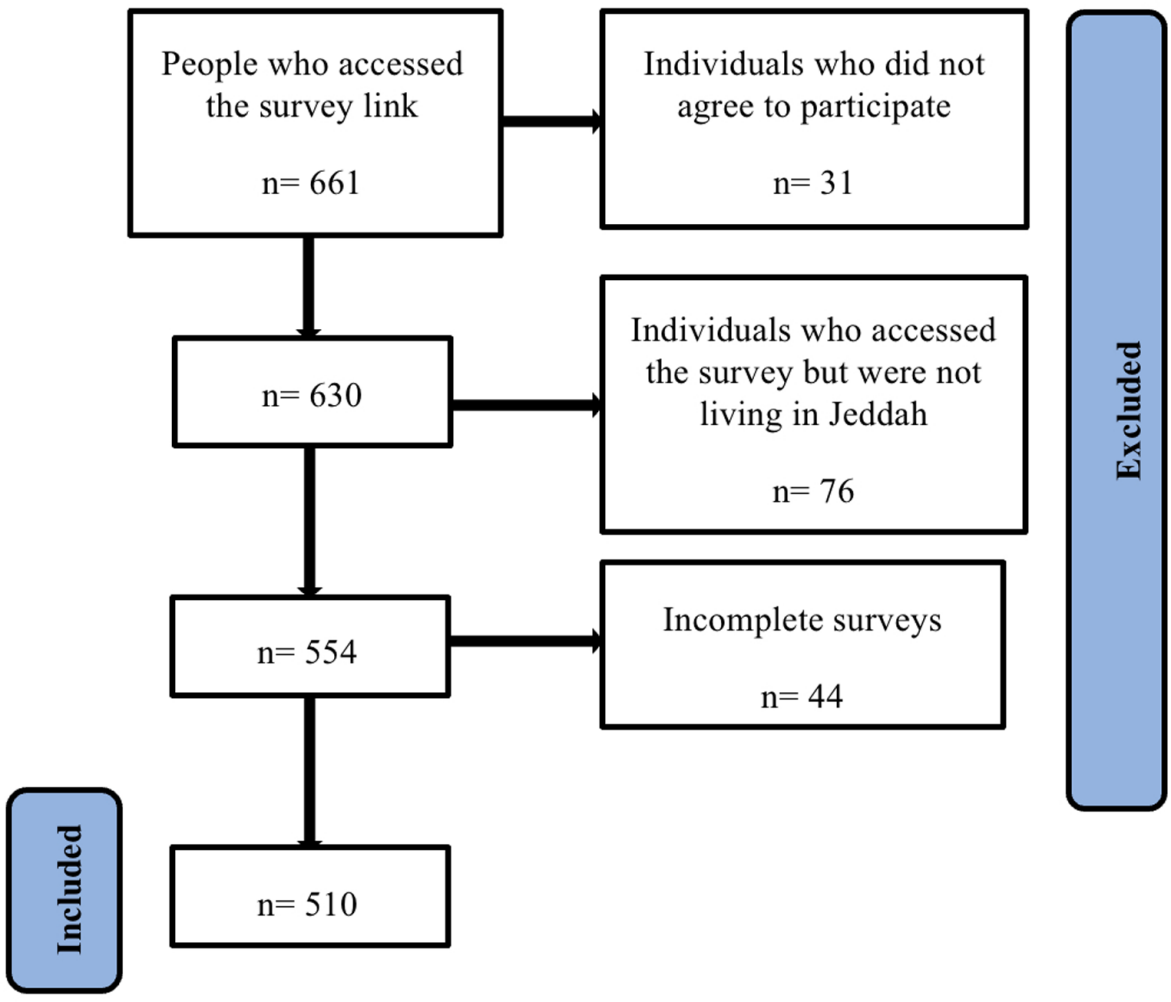
Study subjects.

Initially, our target was to collect 385 complete responses. We expected that both the non-response rate, and the number of incomplete responses to be high, so we decided to distribute the survey to as much people as possible. We closed the survey when we did not get any more responses for one week. After closing the survey, the number of people who accessed it was 661 individuals. After excluding the individuals who did not agree to participate (*n* = 31), those who accessed the survey but were not living in Jeddah (*n* = 76), and incomplete surveys (*n* = 44), we got 510 complete responses, and we included them in the analysis (Figure 1). This exceeded our initial target of 385 responses.

Table 1 shows that more females (54.7%) have responded to the survey compared with men (45.3%). About two thirds of the participants were young adults (<30 years), had a Bachelor degree or higher, were unemployed, and had a middle income (5,000–20,000 SAR/Month). Approximately, half of those who were employed had a job either in the health (*n* = 47) or the educational (*n* = 44) sectors. A large proportion of the participants (68.8%) had a relative in the health sector. Nearly 80% of the respondents had food allergy or knew someone with this condition. The majority of the respondents had no prior experience/training with food allergy through their jobs or work (83.5%).

**Table 1:** Basic characteristics.

Variable	*N* = 510	%
Age group		
18–29	333	65.3
30–44	84	14.4
45–59	73	14.3
60+	20	3.9
Gender		
Male	231	45.3
Female	279	54.7
Highest educational level		
Less than high school	12	2.3
High school	134	26.3
Diploma	44	8.6
Bachelor	283	55.5
High education	37	7.3
Nationality		
Saudi	485	95.1
Non-Saudi	25	4.9
Currently employed		
Yes	196	38.4
No	314	61.6
If employed, sector		
Food	3	1.5
Education	44	22.4
Health	47	23.9
Military	9	4.5
Other	93	47.4
Income		
<5,000	73	14.3
5,000–1,000	119	23.3
11,000–15,000	100	19.6
16,000–20,000	106	20.7
>20,000	112	21.9
Experience/training with food allergy		
Yes	84	16.5
No	426	83.5
Have/Know anyone with food allergy		
Yes	408	80.0
No	102	20.0
Relative in medical field		
Yes	351	68.8
No	159	31.2
Have child <18 years		
Yes	179	35.1
No	331	64.9

### Knowledge of food allergy

The participants answered 56% of the knowledge-based items correctly. We assessed their knowledge of food allergy in 5 major domains: (1) definition and diagnosis, (2) symptoms and severity, (3) triggers and environmental risks, (4) susceptibility and prevalence, and (5) treatment and use of health care. The knowledge of the participants was highest in the symptoms and severity domain (79.9%), while it was weakest in the susceptibility and prevalence domain (37%). Table 2 shows the knowledge score for each domain and its related items. For the definition and diagnosis, even though about half of the participants knew the definition of food allergy, only a small proportion of them (17.1%) could distinguish between food allergy and food intolerance. With regards to symptoms and severity, more than two thirds of the participants (70.2%) knew that food allergy could be fatal. The majority of the participants were aware of all the common symptoms of food allergy except for tongue swelling and trouble breathing (they were not identified by about half the participants). In the triggers and environmental risk domain, only a small proportion of the participants (35.9%) knew that touching an allergenic food may trigger food allergy and that breast feeding might pass allergenic food, consumed by the mother, into children (37.3%). A small proportion of the participants (20.2%) knew that acidic foods (ex: lemons, oranges) do not trigger food allergy. A large proportion of the participants (≥60%) indicated that egg, peanut, or milk is among the 3 most common food allergens in children. On the other hand, less than half of them considered shellfish as the most common food allergen in adults. For the susceptibility and prevalence, a minor proportion of the participants (33.1%) knew that food allergy could be heritable, and that it can go away by age (24.9%). In addition, only half of the participants knew that food allergy is more common in children. Regarding the treatment and use of healthcare, only one third of the participants knew that food allergy is not curable. However, the majority of them (65.6%) were aware that the only way to prevent allergic reactions is to avoid allergenic food.

**Table 2:** Knowledge quiz.

Item	%
	TRUE	FALSE	I don’t Know
Definition and diagnosis	**50**		
An allergic reaction can happen when the body considers a food to be harmful (T)	52.5	19	28.5
Lactose intolerance (trouble digesting dairy products) is the same as having milk allergy (F)	17.1	47.5	35.4
Symptoms and severity	**79**.**9**		
A person can die from having a food allergy reaction (T)	70.2	8.2	21.6
Hives (red bumps or blotches on the skin that can be itchy) are common symptom of a food allergy reaction (T)	88.2	2.4	9.4
Sign of milk allergy reaction			
Hyperactivity (F)	7.7	92.3	–
Hives (T)	84.3	15.7	–
Tongue swelling/trouble breathing (T)	55.5	44.5	–
Stuffy nose (F)	11.4	88.6	–
Triggers and environmental risk	**48**.**5**		
People with food allergies can have an allergic reaction after touching a Food (T)	35.9	36.6	27.5
A person with milk allergy can still drink low-fat milk without having an allergic reaction (F)	6.3	50.2	43.5
Foods eaten by a mother can be passed to her child through her breast milk (T)	37.3	21.2	41.5
Acidic foods (like lemons, oranges, and tomatoes) commonly cause food allergy (F)	20.2	39.6	40.2
3 most common childhood Food Allergies			
Egg	60.0	40.0	–
Milk	61.8	38.2	–
Peanut	61.4	38.6	–
Most common Food Allergy in adults			
Shellfish	42	43.9	14.1
Perceptions of susceptibility and prevalence	**37**		
Allergic diseases run in families (T)	33.1	27.3	39.6
Food allergies can go away as a person gets older (T)	24.9	27.3	47.8
Food allergy is more common in children than adults (T)	52.9	11.4	35.7
Treatment and use of health care	**49**.**6**		
There is a cure for food allergy (F)	30.4	33.5	36.1
The only way to prevent an allergic reaction is to stay away from the food (T)	65.6	16.7	17.7
A person can take a medicine every day to prevent having food allergy reactions (F)	34.6	16.8	48.5

The bold values are the overall score of each given domain of the knowledge quiz.

### The influence of demographical factors on the knowledge of food allergy

Table 3 shows how demographical factors influenced food allergy knowledge among the participants. The data suggest that (1) females, (2) those who were employed, (3) people with a prior experience/training with food allergy through job or work, (4) those with food allergy acquaintance (i.e., have food allergy or know someone with the condition), and (5) those who have relatives working in the medical field had significantly higher food allergy knowledge (*P* < 0.05). In addition, we found that age has a statistically significant influence on the knowledge score of the respondents (F-ratio = 3.335, *P* = 0.019). The results of the *post hoc* test showed that individuals who are 60 years old and above have significantly lower knowledge scores compared with individuals from the other age groups. Regarding the employment sector, individuals who were employed in the health sector had the highest mean knowledge score (M = 48.3, SD = 18.8), yet this finding was not statistically significant (F-ratio = 0.09, *P* = 0.084). Moreover, the influence of some factors, such as educational level, Income, and having school-aged children did not reach a statistical significance (*P* < 0.05).

**Table 3:** Basic characteristics by overall knowledge score.

Variable	Mean (SD)	*P*–value
Age group		
18–29	40.1 (16.4)	0.019
30–44	40.8 (18.0)		
45–59	45.7 (18.1)		
60+^a^	33.9 (16.7)		
Gender		
Male	38.0 (18.3)	0.001
Female	42.9 (15.6)		
Highest educational level		
Less high school	36.3 (23.7)	0.241
High school	41.3 (16.9)		
Diploma	35.6 (15.4)		
Bachelor	41.3 (17.4)		
High education	42.3 (12.9)		
Nationality		
Saudi	40.8 (16.9)	0.958
Non-Saudi	40.6 (19.1)		
Currently employed		
Yes	43.4 (18.3)	0.038
No	39.8 (16.5)		
If employed, sector		
Food	39.3 (18.9)	0.084
Education	43.8 (18.1)		
Health	48.3 (18.8)		
Military	47.6 (12.3)		
Other	39.8 (16.9)		
Income		
<5,000	35.9 (18.0)	0.089
5,000–1,000	42.0 (16.9)		
11,000–15,000	42.8 (16.7)		
16,000–20,000	40.7 (19.1)		
>20,000	40.7 (14.3)		
Experience/training with food allergy		
Yes	46.8 (13.9)	0.001
No	39.5 (17.4)		
Have/Know anyone with food allergy		
Yes	42.2 (16.7)	0.001
No	34.7 (17.3)		
Relative in medical field		
Yes	41.9 (16.2)	0.029
No	38.3 (18.7)		
Have child <18 years		
Yes	41.5 (18.6)	0.521
No	40.5 (16.3)		

^a^ The results of the *post hoc* test showed that respondents aged 60 years and above had significantly lower mean knowledge score compared with respondents from the other age groups.

### Food allergy attitudes and beliefs

We assessed the attitudes of the participants towards (1) the stigma associated with food allergy, (2) the quality of life of allergic patients, (3) current means to treat and manage the condition, (4) policy issues related to the condition. Table 4 shows each one of these domains along with its related items. With regards to stigma and acceptability, even though about two thirds of the participants thought that food allergy is a serious health problem in Saudi Arabia, a small proportion of them agreed that food allergy causes stigma for allergic patients. For instance, only 27.7% of the participants agreed that children with food allergy are bullied at school due to their allergy. In addition, less than half of the respondents (45.9%) agreed that allergic patients are treated differently due to their allergy. For the effect of food allergy on quality of life, most of the participants thought that food allergy has a negative impact on the quality of life of allergic patients. For example, a large proportion of the respondents (73.4%) agreed that allergic patients cannot eat safely at restaurants. Moreover, 71.8% agreed that allergic patients worry a lot about their food allergy. Regarding the treatment and use of health care, less than half of the participants (48.2%) agreed that epinephrine injections are important for the management of severe allergic reactions. For policy Issues, almost all the respondents (93.1%) thought that schools must make policies to keep allergic children safe. When parents of school-aged children were asked further questions regarding policy issues, the majority of them agreed on banning products with nuts (86.1%) and on providing special tables for allergic children at schools (90.1%). Additionally, the participants indicated that finding the cause of food allergy is the most effective way to help allergic patients (Table 5). Furthermore, they mostly preferred to learn about food allergy through internet and/or email (59.0%). Parents of school-aged children chose lectures given by health care providers as the most effective way to learn about food allergy at schools.

**Table 4:** Attitudes and beliefs (Likert scale).

Item	Agree (%)	Neutral (%)	Disagree (%)
Stigma and acceptability			
Food allergy is a serious health problem in Saudi Arabia	61.6	31.3	7.1
People with food allergies are treated differently because of their food allergy	45.9	22.3	31.8
Children with food allergies have overprotective parents	61.9	22.9	15.2
Children with food allergies are teased at school	27.7	22.3	50
Perceptions of quality of life			
For someone who has a food allergy, staying away from the food that he or she is allergic to is difficult	49.2	23.8	27
People who have food allergy worry a lot about their food allergy	71.8	18.6	9.6
It is difficult for people with food allergies to safely eat at restaurants	73.4	16.2	10.4
Treatment and use of health care			
Having injectable epinephrine (EpiPen) is important for most children with severe food allergy	48.2	43.9	7.9
Policy issues			
Schools should have plans for keeping children with food allergies safe at school	93.1	5.5	1.4
Parents with school-aged children (preshool to high school) (*N* = 115) Stigma and acceptability			
Worried if food allergic child play at my house	89.2	4.5	6.3
Policy issues			
Schools should ban all products with nuts	86.1	7.4	6.5
Schools should provide special tables for food allergic children	90.7	3.9	5.4
It would be unfair if my child could not have a peanut butter sandwich because of another student's peanut allergy	57.4	18.3	24.3

**Table 5:** Attitudes and beliefs (MCQs).

Item	*n* (%)
	(*n* = 510)
Most important way to help Food allergy patients	
Develop a cure	155 (30.4)
Improve current treatments	30 (5.9)
Find the cause (s) of Food Allergy	189 (37.1)
Promote school educational programs	32 (6.3)
Promote public awareness campaigns	104 (20.4)
Best way to learn about Food Allergy	
Radio	12 (2.4)
Television	73 (14.3)
Handouts/Brochures	55 (10.8)
Internet/Email	301 (59.0)
Newspapers/Magazines	6 (1.2)
Others	56 (11.0)
NA	7 (1.4)
Parents with school aged children (preschool to high school) (*n* = 115)	
The best way schools can teach parents how to protect their allergic child
Handouts/brochures in the mail	16 (13.8)
Presentation at parent-teacher meetings	1 (0.9)
Parents of food allergic children talking to other parents	9 (7.8)
Doctor or nurse talking about food allergies	84 (73.0)
Others	5 (4.3)

## Discussion

In this study, we assessed food allergy knowledge in 5 areas: (1) definition and diagnosis, (2) symptoms and severity, (3) triggers and environmental risks, (4) susceptibility and prevalence, and (5) treatment and use of health care. We found that the participants' knowledge varied between these areas, with symptoms and severity being the area of strongest knowledge (79.9% of its related items were answered correctly), and susceptibility and prevalence being the area of weakest knowledge (37% of related items answered correctly). In general, the participants were unaware of the absence of cure to food allergy, the role of age in the remission of food allergy, the heritability of the condition, the difference between food allergy and intolerance, the possibility to trigger food allergy by touch, and the transmission of food allergens through breast feeding. Regarding the attitudes, most of the respondents agreed that food allergy negatively impacts the quality of life of allergic patients, and that schools should make policies to protect allergic children. Although nearly 62% of the respondents agreed that food allergy is a serious health problem in Saudi Arabia, they downplayed the stigma associated with this condition. Furthermore, we found that gender, age, prior training with food allergy, food allergy acquaintance (i.e., have food allergy or know someone with the condition), having relatives working in the medical field, and employment status influenced knowledge of food allergy significantly compared with employment sector, educational level, income, and having a school-aged child whose influence did not reach a statistical significance.

### Knowledge of food allergy

Research has shown that people tend to over-report food allergy among themselves because its clinical presentation is similar to some other food related conditions (ex: intolerance, oral allergy syndrome) (3, 4, 15, 16). For example, Gupta et al. found that the prevalence of food allergy using self or parents reporting is 19% and 11.4% among adults and children respectively. However, after further evaluation, the researchers indicated that the convincing food allergy cases are 10.8% and 7.6% for adults and children, respectively (3, 4). Similarly, our data suggest that the participants overestimated food allergy among themselves. Even though 80% of the participants reported food allergy acquaintance (i.e., either have the condition or know someone with it), about 50% of them did not know the difference between milk allergy and lactose intolerance. Overestimation of food allergy might lead to unnecessary avoidance of food, which negatively impacts the quality of life of these individuals. All these findings suggest that access to health care providers is necessary to provide an appropriate diagnosis to patients and to educate them about the difference between food allergy and other forms of adverse food reactions, which could prevent unnecessary avoidance of food for those who do not have food allergy.

Most of the participants (≥60%) thought that milk, peanut, or egg is one of most common childhood food allergens, while 42% considered shellfish as the most common adult food allergen. These allergens, except for egg, are the most common in western countries (3, 4, 10). However, the aforementioned food allergens are not necesserly the most common in other countries because the variation in diets affects how common an allergen is (17). Additionally, because data from the available local studies about common food allergens vary, we could not know whether or not these allergens are the most common locally. For example, Althumiri and colleagues found that the most common food allergens among adults were egg (3.7%), shellfish (3.1%), and peanut (3.0%). On the other hand, milk, egg white, and wheat were reported in another study as the 3 most common food allergens (18, 19). Even though shellfish was not reported as the most common food allergen in the aforementioned studies we considered it as the most common allergen in our study because Jeddah is a coastal city, where seafood is more common in diet compared with most of the other regions of the kingdom. Thus, we expected that shellfish is probably more common than all the other allergens including egg and milk. Also, data regarding common food allergens among children are lacking. Therefore, more studies have to be conducted to identify common food allergens locally, which will benefit allergic patients and will help concerned bodies to pay more attention to these allergens in food labeling.

Less than half the participants (48.2%) in our study were aware of the role of epinephrine injections in managing food-induced anaphylaxis. In addition, other local studies have reported similar results (12, 20). These findings are alarming because epinephrine injections are the only available agents to manage food-induced anaphylactic reactions currently. Research has shown that the increase in epinephrine injections use has caused a plateau in mortality rate related to food-induced anaphylaxis (21–28). Further, poor knowledge of epinephrine injections delays the management of anaphylactic reactions causing an increase in the risk of mortality (25, 26, 29, 30). Sampson et al. found that fatal anaphylactic reactions were associated with delayed use of epinephrine injections for at least 30 min after the onset of anaphylaxis (31). Thus, education campaigns have to be established to increase the awareness of these injections in the management of food-induced anaphylaxis.

Eating food prepared outside home is part of the modern lifestyle that many people follow nowadays (32). Consuming food prepared outside home might increase the risk of having allergic reactions for individuals with food allergy. Pumphrey et al. indicated that 76% of fatal anaphylactic reactions happened upon consuming food outside home (33). Our data suggest that participant's knowledge of triggers and environmental risks of food allergy was low (48.5%) compared with the subjects of a similar study conducted in the US (64.7%) (10). Only about 36% of the respondents knew that touching an allergenic food might trigger food allergy. Nearly half the participants (50.2%) only, knew that low fat milk triggers allergy for individuals with milk allergy. Poor knowledge of food allergy triggers increases the possibility of getting anaphylactic reactions while eating outside home.

### Attitudes towards food allergy

The majority of the participants in our study agreed that food allergy has a negative impact on the quality of life of allergic patients. Nearly 72% agreed that allergic patients are anxious due to their food allergy. Furthermore, 73.4% of the respondents agreed that eating safely at restaurants is difficult for allergic patients. Similarly, other studies indicated that food allergy is costly, and it negatively impacts the quality of life of allergic patients. Regarding the economic burden, Gupta et al. estimated the overall costs on families of allergic children at $20.5 billion annually. Further, they indicated that a large proportion of the money is spent on allergen-free diet (34). Regarding quality of life, Warren et al. found that the constant vigilance due to continuous food allergen avoidance is a major source of stress for allergic patients and their families (35, 36).

Regarding policy issues, the vast majority of respondents (93.1%) agreed that schools need to establish better policies to keep allergic children safe. Additionally, participants with school-aged children further agreed on the implementation of specific policies on schools to protect allergic children. Schools are among the most common sites where anaphylactic reactions occur. It was reported that nearly 18% of children with food allergy had at least 1 allergic reaction at school within 2 years (37). These findings might explain why the majority of participants in our study agreed on establishing policies concerning food allergy at schools.

### The influence of demographical factors on knowledge of food allergy

The data show that having a prior experience with food allergy, food allergy acquaintance, and having a relative in the medical field influenced knowledge score significantly (*P* < 0.05). Likewise, a nationwide study conducted in the US found that the influence of prior food allergy training and food allergy acquaintance on knowledge is statistically significant (10). These findings suggest that targeted educational interventions might have a significant effect in eliminating the knowledge gap among the general population.

Moreover, participants who were 60 years old or more had significantly less knowledge compared with younger participants. We attribute this finding to the high illiteracy rate (60%) among the Saudi population in 1970. In addition, while today's illiteracy rate is only 5.6%, it is believed that the elderly constitutes a majority of that percentage (38).

Furthermore, the influence of educational level on knowledge, surprisingly, was not statistically significant. We attribute this finding to the small sample size included in our study.

## Limitations and future direction

The present study does have some limitations. One of them is limiting the survey distribution to the population of Jeddah, in which case the results from this study cannot be generalized to the whole population of Saudi Arabia. The other limitation is the use of convenient sampling technique in distributing the survey, which could introduce sampling bias. This might affect the representativeness of our sample and, therefore, limit our ability to generalize the results to the entire population of Jeddah. However, due to the scarcity of data regarding food allergy in Saudi Arabia, the present study might be considered as an initial assessment that could help other researchers and experts to start their own investigations regarding food allergy in Saudi Arabia. Therefore, future studies on a nationwide level and with an appropriate sampling technique are recommended.

## Conclusion

In conclusion, our findings demonstrate that the knowledge of food allergy among the members of Jeddah population varied, with strengths and weaknesses identified across different areas. Participants were aware of the severity of food allergy and of its signs and symptoms. However, increased knowledge is required in areas related to the management, the role of age in the remission of food allergy, the heritability of the condition, the difference between food allergy and intolerance, the possibility to trigger food allergy by touch, and the transmission of food allergens through breast feeding. We found that prior training in food allergy, food allergy acquaintance, and having connections with health care workers are associated with significantly higher knowledge of food allergy, which indicates that targeted educational interventions might have a significant effect in eliminating the knowledge gap among the general population.

## Data Availability

The raw data supporting the conclusions of this article will be made available by the authors, without undue reservation.
