# Supplementary material for: Knowledge gaps in food allergy among the general public in Jeddah, Saudi Arabia: Insights based on the Chicago food allergy research survey
Source: Front Allergy. 2022 Dec 23;3:1002694. doi: 10.3389/falgy.2022.1002694 (PMC9816319; doi:10.3389/falgy.2022.1002694)
Supplement: Supplementary file 1 [file Table1.pdf]

# *Supplementary Material*

## 1 Supplementary Tables

### Supplementary Table 1

| Variable | N=510 | % |
|---|---|---|
| Have/Know anyone with food allergy | | |
| Yes | 408 | 80.0 |
| No | 102 | 20.0 |
| If yes, who[a] | | |
| Me* | 5 | 11.4** |
| Friend/ relative | 327 | 80.1 |
| Child (0-18) | 47 | 11.5 |
| Child's classmate or friend | 60 | 14.7 |
| Husband/Wife | 28 | 6.8 |

[a] Participants were allowed to choose more than one answer to this question.

* The number of people who selected this option is relatively low because it was accidentally excluded from the Arabic version of the online survey.

** The number of participants who answered "yes" in the English version was 44, so the percentage was calculated accordingly.
