# Supplementary material for: Knowledge gaps in food allergy among the general public in Jeddah, Saudi Arabia: Insights based on the Chicago food allergy research survey
Source: Front Allergy. 2022 Dec 23;3:1002694. doi: 10.3389/falgy.2022.1002694 (PMC9816319; doi:10.3389/falgy.2022.1002694)
Supplement: Supplementary file 2 [file Datasheet1.pdf]

Questionnaire

Section 1: Demographics

1. Age group, (years):
   A. 18–29
   B. 30–44
   C. 45 –59
   D. 60 and above

2. gender:
   A. Male
   B. Female

3. Highest educational level:
   A. Less than high school
   B. High school
   C. Diploma
   D. Bachelor
   E. Higher education

4. What is your nationality?
   A. Saudi
   B. Non-Saudi

5. Are you currently employed?
   A. Yes
   B. No

6. yes, in which industry are you employed?
   A. Food industry
   B. Education
   C. Health
   D. Military
   E. Others

7. Combined monthly income of household (SAR):
   A. <5000
   B. 5000- 10000
   C. 11000- 15000
   D. 16000- 20000
   E. >20000

8. Have you had any experience or training with food allergy through your job or work?
   a. Yes
   b. No

9. Do you know anyone with a food allergy?
   A. Yes
   B. No

10. If yes, who?
    a. Me*
    b. Child (0-18 years)
    c. Husband/Wife
    d. Friend or relative
    e. Child’s classmate or friend

11. Do you have a relative working in the medical field?
    a. Yes
    b. No

* Note that even though this option is written in the original survey, it was accidentally excluded from the Arabic version of the online form that was distributed to the participants.

Section 2: Knowledge Quiz

12. An allergic reaction can happen when the body considers a food to be harmful.
    A. True
    B. False
    C. I don't know

13. Lactose intolerance (trouble digesting dairy products) is the same as having a milk allergy.
    A. True
    B. False
    C. I don't know

14. A person can die from having a food allergy reaction.
    A. True
    B. False
    C. I don't know

15. Hives (red bumps or blotches on the skin that can be itchy) are common symptom of a food allergy reaction.
    A. True
    B. False
    C. I don't know

16. People with food allergies can have an allergic reaction after touching a food.
    A. True
    B. False
    C. I don't know

17. A person with milk allergy can still drink low-fat milk without having an allergic reaction.
    A. True
    B. False
    C. I don't know

18. Foods eaten by a mother can be passed to her child through her breast milk.
    A. True
    B. False
    C. I don't know

19. Acidic foods (like lemons, oranges, and tomatoes) commonly cause food allergy.
    A. True
    B. False
    C. I don't know

20. Allergic diseases run in families.
    A. True
    B. False
    C. I don't know

21. Food allergies can go away as a person gets older.
    A. True
    B. False
    C. I don't know

22. Food allergy is more common in children than adults.
    A. True
    B. False
    C. I don't know

23. There is a cure for food allergy.
    A. True
    B. False
    C. I don't know

24. The <u>only</u> way to prevent an allergic reaction is to stay away from the food that causes the allergy.
    A. True
    B. False
    C. I don't know

25. A person can take a medicine every day to prevent having food allergy reactions.
    A. True
    B. False
    C. I don’t know

26. Which of the following are the three most common food allergies in children? (Mark three answers)
    A. Egg
    B. Peanut
    C. Wheat
    D. Tree nuts (almonds, walnuts, pecans, cashews)
    E. Milk
    F. Shellfish (shrimp, lobster, crab)

27. Which of the following is the most common food allergy in adults? (Mark one answer)
    A. Milk
    B. Shellfish (shrimp, lobster, crab)
    C. Peanut
    D. I don’t know

28. A boy with a milk allergy accidentally drank some milk. which of the following could be a sign of food allergy reaction? (Mark all that apply)
    A. After 2 days he gets hyperactive and cranky and has headaches
    B. After 15 minutes he gets hives on his face and chest
    C. Immediately his tongue swells and he have trouble breathing
    D. He has a stuffy nose that won’t go away for weeks

Section 3: Attitudes and Beliefs

29. Food allergy is a serious health problem in Saudi Arabia.
    A. Strongly Disagree
    B. Disagree
    C. Natural
    D. Agree
    E. Strongly Agree

30. People with food allergies are treated differently because of their food allergy.
    a. Strongly Disagree
    b. Disagree
    c. Natural
    d. Agree
    e. Strongly Agree

31. Children with food allergies have overprotective parents.
    a. Strongly Disagree
    b. Disagree
    c. Natural
    d. Agree
    e. Strongly Agree

32. Children with food allergies are teased at school.
    a. Strongly Disagree
    b. Disagree
    c. Natural
    d. Agree
    e. Strongly Agree

33. For someone who has a food allergy, staying away from the food that he or she is allergic to is difficult.
    A. Strongly Disagree
    B. Disagree
    C. Natural
    D. Agree
    E. Strongly Agree

34. People who have food allergy worry a lot about their food allergy.
    A. Strongly Disagree
    B. Disagree
    C. Natural
    D. Agree
    E. Strongly Agree

35. It is difficult for people with food allergies to safely eat at restaurants.
    A. Strongly Disagree
    B. Disagree
    C. Natural
    D. Agree
    E. Strongly Agree

36. Having injectable epinephrine (EpiPen) is important for most children with severe food allergies.
    a. Strongly Disagree
    b. Disagree
    c. Natural
    d. Agree
    e. Strongly Agree

37. Schools should have plans for keeping children with food allergies safe at school.
    A. Strongly Disagree
    B. Disagree
    C. Natural
    D. Agree
    E. Strongly Agree

38. Which of the following do you think is the most important to help people with food allergies?
    A. Develop a cure for food allergy
    B. Improve the treatments of food allergy
    C. Find the causes of food allergy
    D. Promote school education programs for food allergy
    E. Promote public awareness campaigns for food allergy

39. Which of the following would be the best way to learn about food allergy?
    A. Radio
    B. Television
    C. Internet/Email
    D. Newspapers/Magazines
    E. Handout/Brochure
    F. Other

40. Do you have children under the age of 18?
    a. Yes
    b. No → end of questionnaire

41. Do your children attend school?
    a. No → end of questionnaire
    b. Yes

42. Which of the following do your children attend? (mark all that apply)
    a. Preschool
    b. Elementary school
    c. Middle school
    d. High school

43. Schools should ban all products with nuts.
    a. Strongly Disagree
    b. Disagree
    c. Natural
    d. Agree
    e. Strongly Agree

44. Schools should have special tables where children with food allergies can safely eat lunch.
    a. Strongly Disagree
    b. Disagree
    c. Natural
    d. Agree
    e. Strongly Agree

45. It would be unfair if my child could not have a peanut butter sandwich because of another student's peanut allergy.
    a. Strongly Disagree
    b. Disagree
    c. Natural
    d. Agree
    e. Strongly Agree

46. I would worry about having a child with food allergy play at my house.
    a. Strongly Disagree
    b. Disagree
    c. Natural
    d. Agree
    e. Strongly Agree

47. What would be the best way for schools to teach parents about how to protect children with food allergies? (mark one answer)
    a. Handouts/brochures in the mail
    b. Presentation at parent-teacher meetings
    c. Parents of food allergic children talking to other parents
    d. Doctor or nurse talking about food allergies
    e. Other

القسم الأول: خصائص العينة

١. العمر (بالسنوات):
أ. ١٨- ٢٩
ب. ٣٠- ٤٤
ج. ٤٥- ٥٩
د. ٦٠ أو أكثر

٢. الجنس:
أ. ذكر
ب. أنثى

٣. المستوى التعليمي:
أ. دون التعليم الثانوي
ب. الثانوية
ج. الدبلوم
د. البكلوريوس
ه. الدراسات العليا (ماجستير فما أعلى)

٤. الجنسية:
أ. سعودي
ب. غير سعودي

٥. هل أنت موظف؟
أ. نعم
ب. لا

٦. في حال أنك أجبت على السؤال السابق ب (نعم)، ما هو مجال عملك:
أ. قطاع الأغذية (المطاعم و مصانع المنتجات الغذائية)
ب. القطاع التعليمي
ج. القطاع الصحي
د. القطاع العسكري
ه. أخرى (حدد المجال ....)

٧. مجموع الدخل الشهري للعائلة (بالريال السعودي):
أ. أقل من ٥٠٠٠
ب. ٥٠٠٠- ١٠٠٠٠
ج. ١١٠٠٠- ١٥٠٠٠
د. ١٦٠٠٠- ٢٠٠٠٠
ه. أكثر من ٢٠٠٠٠

٨. هل تملك خبرة سابقة أو سبق وأن تلقيت دورات تدريبية عن حساسية الطعام؟
أ. نعم
ب. لا

٩. هل لديك معرفة بأشخاص يعانون من حساسية الطعام؟
أ. نعم
ب. لا

١٠. في حال أنك أجبت على السؤال السابق ب (نعم)، فمن هم هؤلاء؟
أ. أنا***
ب. الابن/ة (٠ – ١٨ سنة)
ج. الزوج/ة
د. صديق أو قريب
ه. زميل أو صديق للابن/ة

١١. هل يعمل أي من أقاربك في المجال الصحي؟
أ. نعم
ب. لا

القسم الثاني: اختبار للمعلومات العامة عن حساسية الطعام

١٢. تحدث حساسية الطعام عندما يعتبر الجسم بعض الأطعمة أنها مواد ضارة.
أ. صحيح
ب. خطأ
ج. لا أعلم

١٣. لا يوجد اختلاف بين عسر هضم مشتقات الحليب (عسر هضم اللاكتوز) وحساسية الحليب.
أ. صحيح
ب. خطأ
ج. لا أعلم

١٤. من الممكن أن تتسبب حساسية الطعام في الوفاة.
أ. صحيح
ب. خطأ
ج. لا أعلم

١٥. يعد الشرى أو الطفح الجلدي (بقع منتفخة حمراء على الجلد قد تسبب الحكة) أحد الأعراض الشائعة لحساسية الطعام.
أ. صحيح
ب. خطأ
ج. لا أعلم

ج. لا أعلم

٢٤. يعد تجنب الأطعمة المسببة للحساسية هو السبيل الوحيد لتجنب ظهور أعراض حساسية الطعام.
أ. صحيح
ب. خطأ
ج. لا أعلم

٢٥. تساهم بعض الأدوية في الوقاية من حساسية الطعام شرط أن يتم تناولها بشكل يومي.
أ. صحيح
ب. خطأ
ج. لا أعلم

٢٦. أي من الأطعمة التالية تعد الأكثر تسببا بحساسية الطعام عند الأطفال؟ (اختر ثلاث إجابات)
أ. البيض
ب. القمح
ج: الحليب
د. الفول السوداني
ه. المكسرات (اللوز، عين الجمل، جوز البقان، الكاجو)
و. القشريات البحرية (الروبيان، السلطعون، الاستاكوزا)

٢٧. أي من الأطعمة التالية تعد الأكثر تسببا بحساسية الطعام عند البالغين؟ (اختر إجابة واحدة)
أ. الحليب
ب. الفول السوداني
ج. القشريات البحرية (الروبيان، السلطعون، الاستاكوزا)
د. لا أعلم

٢٨. طفل يعاني من حساسية الحليب وتناول الحليب عن طريق الخطأ. فضلًا اختر الأعراض الدالة على حساسية الطعام مما يلي: (اختر جميع الإجابات التي تعتقد بأنها صحيحة)
أ. الانفعال وفرط في الحركة وصداع بعد يومين.
ب. طفح جلدي في الوجه والصدر بعد ١٥ دقيقة.
ج. تورم في اللسان بجانب ضيق في التنفس مباشرة.
د. انسداد في الأنف لأسابيع.

القسم الثالث: سلوكيات أفراد المجتمع تجاه حساسية الطعام

٢٩. تعتبر حساسية الطعام مشكلة صحية جدية في المملكة العربية السعودية.

١٦. من الممكن أن تظهر أعراض حساسية الطعام عند الأشخاص المصابين بمجرد لمس الطعام.
أ. صحيح
ب. خطأ
ج. لا أعلم

١٧. يستطيع الأشخاص المصابون بحساسية الحليب شرب الحليب قليل الدسم دون أن يتسبب ذلك في ظهور أعراض الحساسية.
أ. صحيح
ب. خطأ
ج. لا أعلم

١٨. يمكن للأطعمة التي تسبب الحساسية التي تستهلكها الأم أن تنتقل لطفلها من خلال حليب الأم خلال الرضاعة.
أ. صحيح
ب. خطأ
ج. لا أعلم

١٩. الأطعمة الحمضية (مثل الليمون والبرتقال والطماطم) تعد من الأطعمة الشائعة المسببة للحساسية.
أ.صحيح
ب. خطأ
ج. لا أعلم

٢٠. حساسية الطعام تعد وراثية.
أ. صحيح
ب. خطأ
ج. لا أعلم

٢١. يمكن أن يتعافى الانسان من حساسية الطعام مع تقدم العمر.
أ. صحيح
ب. خطأ
ج. لا أعلم

٢٢. تعد حساسية الطعام أكثر شيوعًا بين الأطفال مقارنةً البالغين.
أ. صحيح
ب. خطأ
ج. لا أعلم

٢٣. يمكن استخدام الأدوية لعلاج حساسية الطعام بشكل دائم.
أ. صحيح
ب. خطأ

أ. أوافق بشدة
ب. موافق
ج. محايد
د. غير موافق
ه. غير موافق بشدة

٣٠. يُعامَل الأشخاص ممن يعانون من حساسية الطعام بطريقة مختلفة نظرًا لحساسية الطعام المصابين بها.
أ. أوافق بشدة
ب. موافق
ج. محايد
د. غير موافق
ه. غير موافق بشدة

٣١. لدى الأطفال المصابين بحساسية الطعام أولياء أمور مفرطون في حمايتهم.
أ. أوافق بشدة
ب. موافق
ج. محايد
د. غير موافق
ه. غير موافق بشدة

٣٢. يعاني الأطفال المصابين بحساسية الطعام من التنمر في المدرسة.
أ. أوافق بشدة
ب. موافق
ج. محايد
د. غير موافق
ه. غير موافق بشدة

٣٣. من الصعب للمصابين بحساسية الطعام تجنب الأطعمة المسببة للحساسية.
أ. أوافق بشدة
ب. موافق
ج. محايد
د. غير موافق
ه. غير موافق بشدة

٣٤. يعاني الأشخاص المصابون بحساسية الطعام من ارتفاع مستوى القلق.
أ. أوافق بشدة
ب. موافق
ج. محايد
د. غير موافق
ه. غير موافق بشدة

٣٥. يصعب تناول الطعام الآمن خارج المنزل على الأشخاص الذين يعانون من حساسية الطعام.
أ. أوافق بشدة
ب. موافق
ج. محايد
د. غير موافق
ه. غير موافق بشدة

٣٦. يعد استخدام حقنات الأدرينالين ضروريا للتعامل مع حالات حساسية الطعام المفرطة.
أ. أوافق بشدة
ب. موافق
ج. طبيعي
د. غير موافق
ه. غير موافق بشدة

٣٧. ينبغي على المدارس أن تتبنى خططًا لحماية الأطفال المصابين بحساسية الطعام داخل المدرسة.
أ. أوافق بشدة
ب. موافق
ج. محايد
د. غير موافق
ه. غير موافق بشدة

٣٨. أي من الإجراءات التالية تعتقد أنه الأكثر فاعلية في مساعدة الأشخاص الذين يعانون من حساسية الطعام؟
ا. تطويرعلاج دائم لحساسية الطعام
ب. تطوير العلاجات المتاحة لعلاج حساسية الطعام
ج. تحديد العوامل المسببة لحساسية الطعام
د. تنظيم برامج مدرسية توعوية عن حساسية الطعام
هـ. تنظيم حملات توعوية لأفراد المجتمع عن حساسية الطعام

٣٩. أي مما يلي يعد أفضل وسيلة للتعلم عن حساسية الطعام؟
أ. المذياع (الراديو)
ب. التلفاز
ج. الإنترنت/البريد الإلكتروني
ه. الجرائد/ المجلات
و. البروشورات/النشرات التوعوية
ز. أخرى

٤٠. هل لديك أطفال تحت سن الثامنة عشر؟
أ. نعم
ب. لا (نهاية الاستبيان)

ه. غير موافق بشدة

٤٧. أي من الآتي تعتقد أنه الأكثر فاعلية للمدارس لتوعية أولياء الأمور حول حماية أطفالهم المصابين بحساسية الطعام
أ. البروشورات/النشرات التوعوية
ب. العروض التقديمية في اجتماع أولياء الأمور
ج. الحديث مع أولياء أمور لديهم أطفال مصابون بحساسية الطعام
د. استضافة ممرض أو طبيب للحديث عن حساسية الطعام
ه. أخرى

٤١. هل يذهب أطفالك للمدرسة؟
أ. نعم
ب. لا (نهاية الاستبيان)

٤٢. في أي مرحلة تعليمية يعد أطفالك ؟
أ. الروضة
ب. المرحلة الابتدائية
ج. المرحلة المتوسطة
د. المرحلة الثانوية

٤٣. يجب على المدارس منع جميع المنتجات التي تحوي المكسرات:
أ. أوافق بشدة
ب. موافق
ج. محايد
د. غير موافق
ه. غير موافق بشدة

٤٤. يجب على المدارس توفير طاولات مخصصة للأطفال المصابين بحساسية الطعام لتناول الطعام بطريقة آمنة:
أ. أوافق بشدة
ب. موافق
ج. محايد
د. غير موافق
ه. غير موافق بشدة

٤٥. من غير العادل ألا يتناول طفلي ساندوتش الفول السوداني بسبب حساسية طفل آخر تجاه الفول السوداني.
أ. أوافق بشدة
ب. موافق
ج. محايد
د. غير موافق
ه. غير موافق بشدة

٤٦. سأكون قلقًا إن كان هنالك طفل في منزلي يعاني من حساسية الطعام
أ. أوافق بشدة
ب. موافق
ج. محايد
د. غير موافق
